# Apolipoprotein M inhibits proliferation and migration of larynx carcinoma cells

**DOI:** 10.1038/s41598-020-76480-w

**Published:** 2020-11-10

**Authors:** Haixiang Xue, Miaomei Yu, Ying Zhou, Jun Zhang, Qinfeng Mu, Tongbing Chen, Guanghua Luo, Jisheng Liu

**Affiliations:** 1 Department of Otorhinolaryngology, The Third Affiliated Hospital of Soochow University, Changzhou, Jiangsu Province China; 2 Comprehensive Laboratory, The Third Affiliated Hospital of Soochow University, 185 Juqiang St, Changzhou, Jiangsu Province China; 3 Department of Pathology, The Third Affiliated Hospital of Soochow University, Changzhou, Jiangsu Province China; 4 Department of Otorhinolaryngology, The First Hospital Affiliated to Soochow University, 188 Shizi St, Suzhou, Jiangsu Province China

**Keywords:** Cancer, Cell biology, Oncology

## Abstract

Prior studies have shown that apolipoprotein M (APOM) is involved in the development of some cancers. Here we investigated the effects of APOM on larynx cancer (LC). 20 patients with vocal cord polyps and 18 patients with LC were included in this study. The protein and mRNA levels of the samples were analysed using the Wes-ProteinSimple system (or traditional Western blot) and PCR technology, respectively. APOM protein level in cancer tissues was lower than that in paracarcinomatous (*P* = 0.0003) and polyp tissues (*P* < 0.0001). APOM overexpression significantly inhibited TU686 cell proliferation (*P* < 0.0001) and migration (*P* < 0.01), and increased expression of vitamin D receptor (VDR, *P* < 0.0001) as well as nuclear factor erythroid 2-like 3 (NFE2L3, *P* = 0.0215). In addition, matrix metalloproteinase-10 (MMP-10) mRNA level was significantly reduced in the APOM overexpression group (*P* = 0.0077). However, Western blot analysis showed that APOM overexpression did not change VDR, NFE2L3 and MMP-10 protein levels (*P* > *0.05*). In summary, APOM inhibits the proliferation and migration of LC cells, but may not be related to VDR, NFE2L3 and MMP-10, which needs further study.

Larynx cancer (LC) is the second most common head and neck cancer in the world. In 2018, the number of new LC cases increased to 177,422 worldwide. The incidences of this carcinoma in Asia and Europe are 52.6% and 22.5%, respectively^1^. In head and neck cancer, the high incidence of LC is mainly attributed to tobacco and alcohol consumption, which are recognized risk factors for LC. In addition, the prevalence of LC in men is approximately 5 times that in women^2^. The larynx is a reticular structure composed of cartilage, ligaments, membranes and internal and external laryngeal muscles. Clinically, the larynx is divided into 3 subregions including the supraglottis, glottis and subglottis. The most common site of LC is the glottis, followed by the supraglottis and subglottis sites. Squamous cell carcinoma (SCC) is the most common histology type of LC, and 95% of laryngeal SCC originates from the stratified squamous epithelium of the larynx^3^.

Lipid metabolism is essential for tumour progression, and its related proteins can be used as therapeutic targets for tumours^4^. Studies have shown that serum high-density lipoprotein cholesterol (HDL-C) levels may be positively or negatively correlated with cancer morbidity and mortality^5^. The expression of apolipoprotein A-I, the main component of HDL, has been shown to be upregulated in LC tissues^6^, but downregulated in the plasma of patients with LC^7^. However, there is no clear evidence that other apolipoproteins are related to the occurrence and development of LC.

Apolipoprotein M (APOM), a member of the lipocalin superfamily and a carrier of sphingosine 1-phosphate (S1P)^8^, was first discovered and isolated from chylomicron emulsions by Xu N et al. in 1999^9^. APOM, like other apolipoproteins, is involved in lipid transport and metabolism. Our previous studies have shown that APOM is also involved in the development of several cancers. For example, in 2010, we found that colorectal cancer patients with lymph node metastasis expressed significantly high levels of APOM mRNA^10^. Additionally, in 2017, we also found that APOM increased the levels of vitamin D receptor (VDR) in colorectal cancer cells, suggesting that APOM may play an antineoplastic role by upregulating the expression of VDR^11^. In 2018, our research demonstrated that APOM can promote the proliferation and invasion of non-small cell lung cancer cells, which is related to the upregulation of S1P-receptor 1 and activation of the ERK1/2 and PI3K/AKT signalling pathways induced by APOM^12^. Furthermore, we reported that APOM can suppress the proliferation and invasion of SMMC7721 cells, a hepatocellular carcinoma line, through VDR pathways^13^. In the present study, we compared the expression of APOM in different types of laryngeal tissues, analysed the biological effects of APOM overexpression on LC cells, and explored the possible mechanisms of APOM in the development of LC.

## Materials and methods

The study was approved by the ethics committee of the Third Affiliated Hospital of Soochow University. All patients provided written informed consent and all methods were performed in accordance with relevant guidelines and regulations.

### Patients and samples

20 patients (12 male and 8 female patients, aged from 39 to 67 years) with vocal cord polyps and 18 patients (all male patients, aged from 55 to 78 years) with LC were included in this study. 20 vocal cord polyp tissues, 18 LC tissues and 18 matched paracarcinomatous tissues were collected by surgical resection. The tumour tissues and paracarcinomatous tissues were frozen in liquid nitrogen immediately after resection for further experiments. Parts of the specimens were fixed in 10% (V/V) formalin and embedded in paraffin for immunohistochemistry analyses.

### Protein analysis using the Wes-Protein simple system

Total protein was extracted from the vocal cord polyps, LC tissues and matched paracarcinomatous tissues using a total protein extraction kit (BestBio, Shanghai, China) in accordance with the manufacturer’s instructions. Cytoplasmic and nuclear proteins from cultured cells were purified with NE-PER Nuclear and Cytoplasmic Extraction Reagents (Pierce Biotechnology, Rockford, USA). The protein concentration was assayed using the BCA Protein Assay kit (BestBio, Shanghai, China). A western system (Wes-ProteinSimple, USA) based on capillary electrophoresis technology was used to quantify the levels of APOM (mouse anti-APOM, 1:75, Abonva, Taiwan) and β-actin (rabbit anti-β-actin, 1:50, CST, USA). Lamin B1 (rabbit anti-Lamin B1, 1:50, Beyotime, China) was selected as the internal control of nuclear protein. The protein levels of VDR (rabbit anti-VDR, 1:1000, Abcam, USA), NFE2L3 (rabbit anti-NFE2L3, 1:200, Novus, USA) and MMP-10 (mouse anti-MMP-10, 1:500, R&D, USA) were detected by traditional Western blot technology, and β-actin (rabbit anti-β-actin, 1:1000, CST, USA) was used as internal control.

### Immunohistochemistry

For immunohistochemistry analyses, 3-µm thick paraffin-embedded tissues were dewaxed in xylene, rehydrated with graded ethanol, and washed in 0.1 M Tris–HCl (pH 7.6). Antigen retrieval was carried out in a 10 mmol/L citrate buffer (pH 6.0) at 100 °C for 30 min. A 0.3% hydrogen peroxide solution was used to block endogenous peroxidase activity for 15 min. After rinsing in phosphate buffer saline (PBS) for 5 min, nonspecific binding of the primary antibody was eliminated by incubation with 5% bovine serum albumin for 15 min at room temperature. The sections were incubated with a mouse anti-human antibody against APOM (1:200, Abnova, Taipei, Taiwan) at 4 °C overnight. After washing again, the sections were then incubated with horseradish peroxidase-conjugated goat anti-mouse immunoglobulin (Dako, Glostrup, Denmark) for 2 h at room temperature. Sections without primary antibody were directly incubated with the secondary antibody as a control. Peroxidase activity and haematoxylin–eosin (HE) staining were performed with a commercial visualization system according to the manufacturer’s instructions (Dako) and standard procedures, respectively.

### Cell culture

The human laryngeal squamous carcinoma cell line TU686 was purchased from BNBio Tech Co., Ltd., China. Cells were cultured in RPMI 1640 medium (Gibco, Life Technologies, NY, USA) supplemented with 10% foetal bovine serum (FBS) (Gibco, Life Technologies, Melbourne, Australia), 100 U/mL penicillin and 100 μg/mL streptomycin (Gibco, Life Technologies, NY, USA), and incubated at 37 °C in a humidified atmosphere with 5% CO_2_. When the cells reached approximately 90% confluence, they were washed twice with PBS and digested with 0.25% trypsin/0.53 mM EDTA (Gibco, Life Technologies). The cell suspension was collected and centrifuged at 1000 rpm for 3 min. After centrifugation, the cells were resuspended with fresh complete medium and seeded in suitable culture vessels for continued culture. For cryopreservation, the cells were suspended in pre-cooling RPMI 1640 medium containing 10% dimethyl sulfoxide (DMSO) (Sigma-Aldrich, St. Louis, USA) and 40% FBS, transferred to cryotubes, cooled from 4 °C to − 80 °C at a rate of 1 °C/min in a Nalgene container and stored in liquid nitrogen for long-term preservation.

### Lentivirus transfection

Lentiviruses carrying the APOM gene or empty vector (as a negative control, NC) were constructed by GeneChem Co., Ltd. (Shanghai, China). TU686 cells were seeded in 6-well plates at a density of 1 × 10^5^ cells/well and incubated for 24 h to reach 50% confluence. The medium was then replaced with a medium containing the lentivirus at a multiplicity of infection (MOI) of 10 plaque-forming units/cell according to the manufacturer’s instructions. After 72 h, cells successfully infected with the lentivirus presented positive green fluorescent protein (GFP) and green fluorescence could be observed under fluorescence microscopy (Olympus, Tokyo, Japan). The definite efficiency of APOM overexpression was evaluated by quantitative real-time PCR (qPCR).

### RNA extraction and reverse transcription

A Total RNA Purification Kit (Biocolor, Shanghai, China) was used to extract total RNA from the cell lines and sample tissues following the manufacturer’s instructions. The concentration and purity of the RNA were determined by measuring the absorbance at 260/280 nm using a BioPhotometer (Eppendorf, Germany), and a ratio of 1.8 to 2.0 indicated high purity. cDNA, the reverse transcription product of total RNA, was obtained by the RevertAid First Strand cDNA Synthesis kit (Thermo Scientific).

### Quantitative real-time PCR

All qPCR assays were performed on a LightCycler 480 II system (Roche Diagnostics GmbH, Mannheim, Germany). The PCR reaction system and conditions were carried out in accordance with our previous study^11^. The mRNA levels of APOM, VDR, nuclear factor erythroid 2-like 3 (NFE2L3) and matrix metalloproteinase-10 (MMP-10) were normalized to the mRNA level of glyceraldehyde-3-phosphate dehydrogenase (GAPDH) by comparative threshold cycling (2^−ΔCt^)^14^ for quantification. The primer and probe sequences are provided in Table 1.

**Table 1:** Sequences of the primers and probes used in this study.

Gene	Primer/Probe	Sequences (5′ → 3′)
APOM	Forward primer	CTGACAACTCTGGGCGTGGAT
	Reverse primer	TGTCCACAGGGTCAAAAGTTGC
	Probe	FAM-AGTTCCCAGAGGTCCACTTGGGCCA-BHQ1
VDR	Forward primer	GCTAAGATGATACCAGGATTCAGAGAC
	Reverse primer	AAGGACTCATTGGAGCGCAAC
	Probe	FAM-ACCTCTGAGGACCAGATCGTACTGCTGA-BHQ1
NFE2L3	Forward primer	TCAGCAGAATGATGATGATGAAAAC
	Reverse primer	GCTGTGATGAAAGCAACTGGAAT
	Probe	FAM-AATAGCAGAGAAACCTGACTGGGAGGC-BHQ1
MMP-10	Forward primer	TCGCCCAGTTCCGCCTT
	Reverse primer	GCACCAGGGGTTCCTCAGTAG
	Probe	FAM-TGGCATTCAGTCTCTCTACGGACCTC-BHQ1
GAPDH	Forward primer	CAGGGCTGCTTTTAACTCTGGT
	Reverse primer	CATGGGTGGAATCATATTGGAAC
	Probe	Cy5-TGGATATTGTTGCCATCAATGACCCCT-BHQ2

### Cell proliferation assay

The TU686 cell proliferation assay was performed with a Cell Counting Kit-8 (CCK-8) (Dojindo, Kumamoto, Japan) according to the manufacturer’s instructions. Cells were seeded in 96-well plates at a density of 5 × 10^4^ cells/mL, with six replicate wells in each group. After 24 h of culture, 10 µL CCK-8 was added to each well and incubated for 3.5 h, and then the optical density (OD) value was measured at a wavelength of 450 nm. The same test was repeated at 48 h and 72 h.

### Cell migration assay

Cells from the APOM-OE and NC groups were seeded in 6-well plates at a density of 5 × 10^5^ cells/mL and cultured until confluency. After washing with PBS, the cells were cultured in RPMI 1640 medium containing 5% FBS for 24 h. After discarding the medium from each well, several parallel lines were drawn on the bottom of 6-well plates. The wounds, perpendicular to the bottom horizontal lines, were scratched by sterile 200 μL pipette tips. The suspended cells were washed with PBS and replaced with fresh serum-free medium containing 1% bovine serum albumin. The wound distances were determined using an inverted microscope (Olympus CKX41, Tokyo, Japan) and a digital camera (Olympus, Japan) at 0 h, 24 h, 48 h and 72 h, and the percentage of wound healing was further analysed.

### Statistical analysis

Statistical analyses were performed using GraphPad Prism 8.0 software (GraphPad Prism Software Inc., San Diego, CA, USA). The mean ± standard deviation or the median with interquartile range represents the data concentration trends. Significant differences between two groups were determined by Student’s *t*-test. Wilcoxon matched-pairs signed rank test was used to compare the differences in APOM protein levels between the LC and matched paracarcinomatous tissues. One-way ANOVA was used for the multiple comparisons and a *P* value less than 0.05 was considered statistically significant.

## Results

### APOM protein levels in vocal cord polyps, LC tissues and matched paracarcinomatous tissues

**Figure 1:**
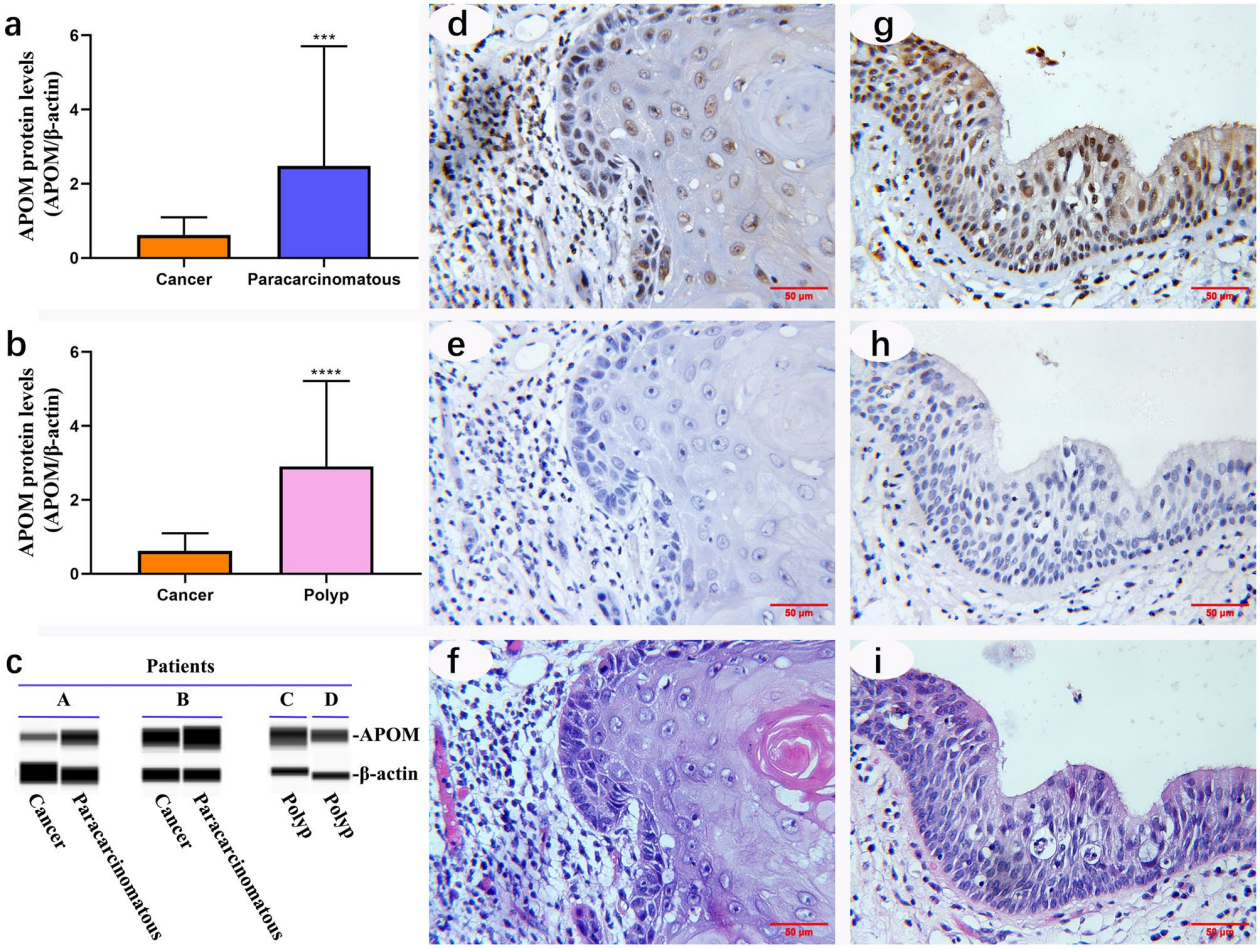
APOM protein levels in vocal cord polyps, LC tissues and matched paracarcinomatous tissues. The APOM protein levels (**a–c**) in different types of laryngeal tissues were detected by the Wes-ProteinSimple system (median with interquartile range) and full-length blots are presented in Supplementary Figure S1. 3 consecutive sections of laryngeal carcinoma tissue (**d–f**), 3 consecutive sections of paracarcinomatous tissue (**g–i**), the anti-APOM antibody-positive tissues (**d** and **g**), the anti-APOM antibody NCs (**e** and **h**) and the H&E staining results (**f** and **i**). Scale bar = 50 μm.

The results of western blot based on capillary electrophoresis technology showed that the APOM protein level in cancer tissues was lower than that in paracarcinomatous tissues (*P* = 0.0003) (Fig. 1a and c) and in vocal cord polyp tissues (*P* < 0.0001) (Fig. 1b and c). Data of all samples detected by capillary western analyses were provided in Supplementary Figure S1. The immunohistochemistry results indicated that the APOM protein level in LC tissues was lower than that in paracarcinomatous tissues (Fig. 1d and g). The negative controls of the anti-APOM antibody were shown in Fig. 1e and h. The H&E staining results of LC and paracarcinomatous tissues were presented in Fig. 1f and i.

### Effects of APOM overexpression on the proliferation and migration of TU686 cells

**Figure 2:**
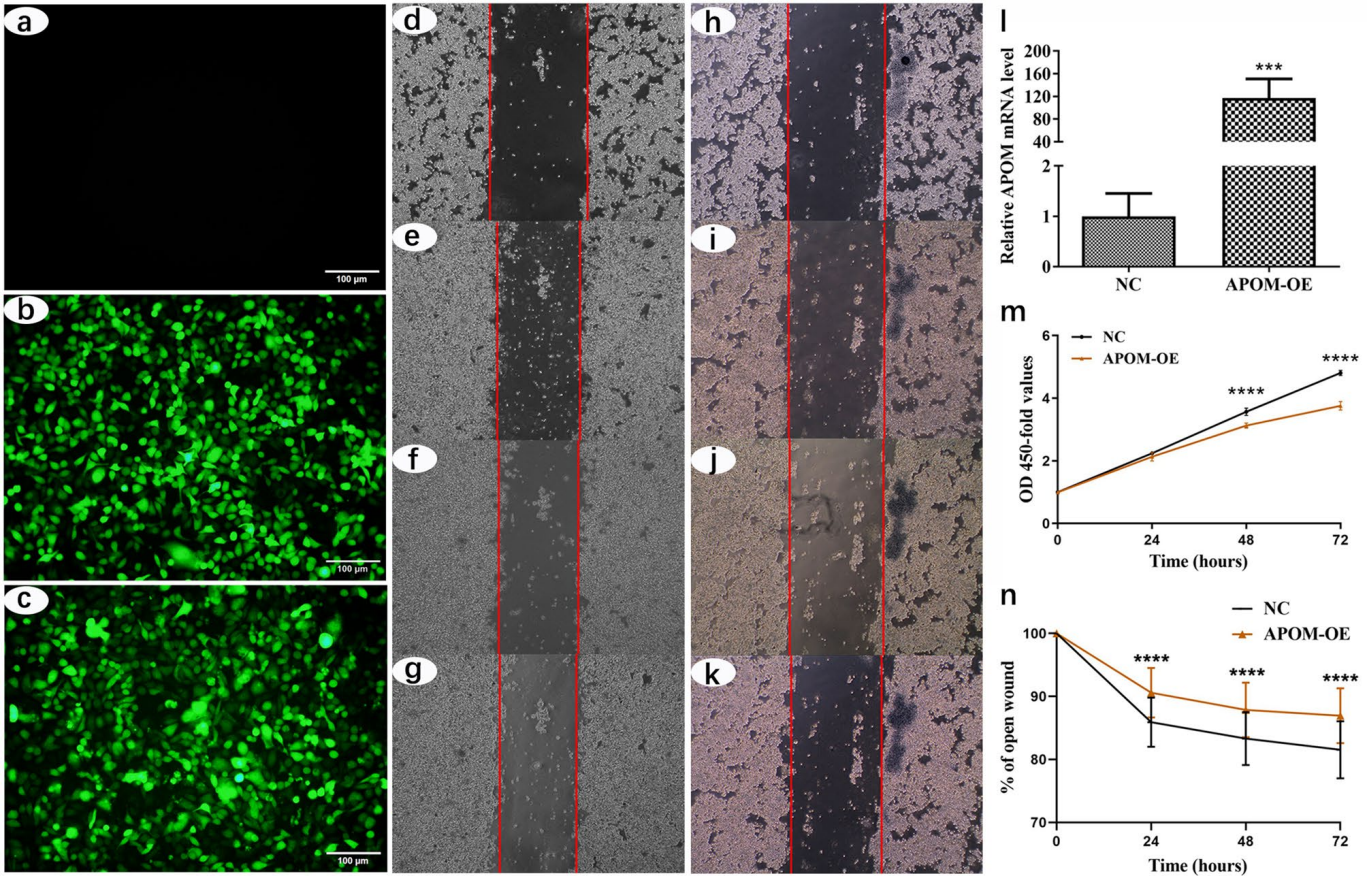
Effects of APOM overexpression on the proliferation and migration of TU686 cells. Fluorescence micrographs of TU686 cells are shown in (**a–c**): lentivirus negative group (**a**), lentivirus carrying empty vector group (**b**) and lentivirus carrying APOM gene group (**c**). Panel **l** shows the efficiency of the APOM overexpression (mean ± standard deviation). The effect of APOM on the proliferation of TU686 cells is shown in panel **m** (mean ± standard deviation). The effect of APOM on the migration of TU686 cells is shown in panels d to k and **n** (mean ± standard deviation).

GFP-labelled lentivirus vectors were successfully transfected into TU686 cells. At 72 h post-transfection, the transfection efficiency was more than 70% by observing the percentage of GFP-positive cells under a fluorescent microscope (Fig. 2a–c). Figure 2l indicates that the APOM mRNA level in APOM-OE TU686 cells was significantly higher (approximately 120-fold) than that in the NC group of cells. In addition, protein analysis results indicated that there are distinct APOM overexpression bands in APOM-OE group, but not in NC group (Supplementary Figure S2). CCK-8 assay showed that APOM overexpression significantly inhibited cell proliferation at 48 h (*P* < 0.0001) and 72 h (*P* < 0.0001) (Fig. 2m). The wound healing assays (Fig. 2d–k and n) showed that the cell migration rates of the APOM-OE group were significantly lower than those of the NC group at 24 h, 48 h and 72 h (*P* < 0.0001).

### Effects of APOM on the expression of VDR, NFE2L3 and MMP-10

**Figure 3:**
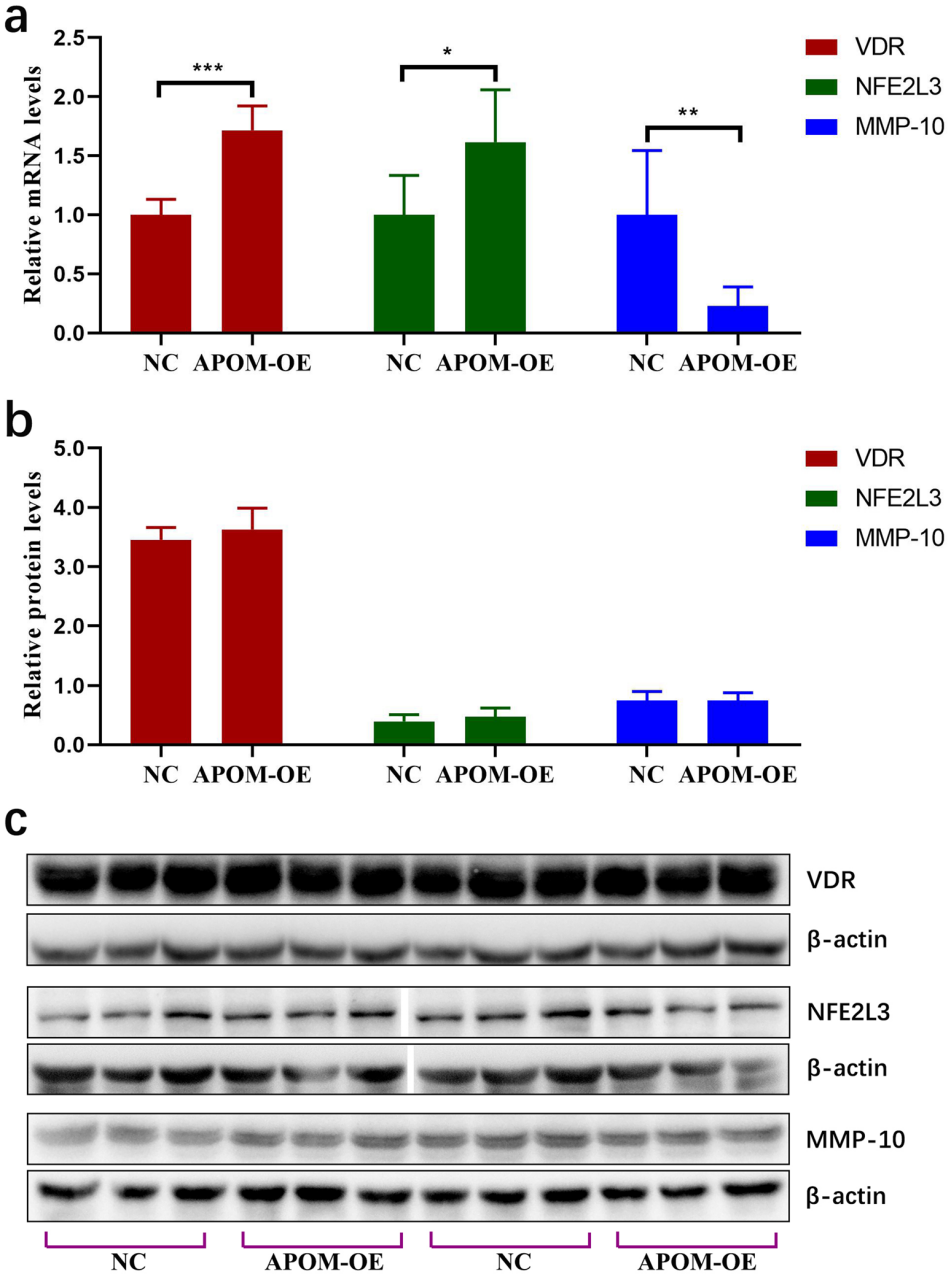
Effects of APOM on the expression of VDR, NFE2L3 and MMP-10. Effect of APOM on the mRNA levels (**a**) and protein levels (**b** and **c**) of VDR, NFE2L3 and MMP-10. Full-length blots are presented in Supplementary Figure S3.

The mRNA levels of VDR, NFE2L3 and MMP-10 were detected in the APOM-OE and NC groups. Figure 3a shows that the mRNA levels of VDR and NFE2L3 in the APOM-OE group were 1.72-fold (*P* < 0.0001) and 1.62-fold (*P* = 0.0215) higher than those in the NC group, respectively. The mRNA level of MMP-10 in the APOM-OE group was only 23% of that in the NC group (*P* = 0.0077). However, Western blot analysis (Fig. 3b,c and Supplementary Figure S3) showed that APOM overexpression did not change VDR, NFE2L3 and MMP-10 protein levels (*P* > 0.05).

## Discussion

Recent studies have investigated whether APOM is involved in the occurrence and development of tumours, but the conclusions have been inconsistent. According to reports, APOM may exert anti-tumour effects by upregulating VDR expression in colon cancer cells^11^, while in non-small cell lung cancer, APOM may promote tumour cell proliferation and invasion by upregulating S1PR1 and activating the ERK1/2 and PI3K/AKT signalling pathways^12^. In the present study, we first found that the APOM protein level in LC tissues was significantly lower than that in paracarcinomatous tissues and vocal cord polyp tissues. These results suggest that APOM may be related to the carcinogenesis of LC.

To explore the effect of APOM on the biological function of LC, we constructed and transfected lentiviral vectors to overexpress APOM in TU686 cells. The results revealed that overexpression of APOM inhibited cell proliferation and migration, which can be concluded that APOM might inhibit the development of LC. As a secretory protein, APOM is secreted into the cell culture medium to function. Changing the medium daily may attenuate the effect of APOM overexpression. Therefore, it is not recommended to replace medium during migration assay. In addition, in order to avoid the effect of APOM in serum on cell migration, the medium must be replaced with serum-free medium containing albumin after scratching.

APOM is the physiological carrier of sphingosine 1-phosphate (S1P) in the blood circulation, and plays a role in protecting endothelial cells through the S1P axis^8^. Although APOM is a secreted protein, it is also usually positive in the cytoplasm, as we have previously studied the localization of APOM in colorectal cells^10^. In this study, APOM was positive in the nucleus (Fig. 1d and g), which is really puzzling. To explore the reason, we separated and prepared cytoplasmic and nuclear extracts from cultured TU686 cells transfected with lentiviral vectors. Protein analysis showed that the overexpressed APOM mainly existed in the cytoplasm, and a small amount existed in the nucleus (Supplementary Figure S2b and S2c). However, endogenous APOM was not evident in cytoplasmic and nuclear extracts compared to total protein fraction (Supplementary Figure S2a). The probable reason was that some endogenous APOM was lost during the separation and preparation of cytoplasmic and nuclear proteins. Another important reason is that the final concentration of extracted nuclear protein is low, the maximum loading concentration is only 0.65 µg/µL, while the concentration of total protein and cytoplasmic protein is 1.5 µg/µL. It is noteworthy that there are more APOM antibody positive bands in the region of molecular weight greater than 40 kDa, whether in cytoplasmic proteins or nuclear proteins. It is not clear whether these bands are APOM polymers or non-specific bands. In summary, endogenous APOM existed in TU686 cells, but it could not be distinguished whether it was mainly distributed in the cytoplasm or in the nucleus. Therefore, more experiments are needed to reveal the mystery of APOM localization in the nucleus of laryngeal tissues.

Next, we investigated the effect of APOM overexpression on the mRNA and protein levels of VDR, NFE2L3 and MMP-10 to further study the possible mechanism by which APOM inhibits the development of LC. Many studies have shown that VDR plays an important role in cell proliferation, differentiation, angiogenesis and apoptosis. Our previous research found that APOM could upregulate the VDR mRNA level in colorectal cancer cells^11^, and APOM could also affect the biological functions of SMMC772I cells via VDR signalling, such as inhibiting proliferation and metastasis^13^. Thus, we examined the VDR mRNA and protein levels in the TU686 cells of the APOM-OE group and NC group. The results showed there was a significant difference in VDR mRNA expression between the two groups, but no significant difference in protein levels, suggesting inhibitory effects induced by APOM on LC cells may not be related to VDR signal transduction.

NFE2L3 is the transcription factor NRF3 and belongs to the cap “N” collar family^15^. NFE2L3 is highly expressed in colorectal adenocarcinoma^16^. Moreover, NFE2L3 is involved in regulating the growth and metastasis of tumours, such as thyroid^17^, pancreatic^18^ and breast carcinomas^19^. Intriguingly, the effects of NFE2L3 on various cancers are very different. NFE2L3 promotes the proliferation of colorectal cancer cells by activating the 20S proteasome and degrading the tumour suppressor p53, which is associated with the poor prognosis of colorectal cancer^20^. In breast cancer, NFE2L3 not only reduces the number of G0/G1 phase cells but also increases the number of S and G2/M phase cells, thus inhibiting the proliferation of breast cancer cells^19^. NFE2L3 also blocks the metastasis of breast cancer cells through the AKT/ID3 axis^19^. In this study, although NFE2L3 mRNA level in the APOM-OE group was higher than that in the NC group, there was no significant difference in protein levels between the two groups, suggesting that NFE2L3 may not be involved in the development of LC induced by APOM.

MMP-10, also known as stromelysin, is a tumour-associated gene. It has the ability to hydrolyse extracellular matrix proteins, such as collagen type III, IV, and V, elastin, proteoglycans and glycoproteins. MMP-10 is involved in the angiogenesis process^21^ and can promote cancer cell invasion and metastasis^21,22^. The present study found that APOM significantly inhibited MMP-10 mRNA expression, but did not affect the expression of MMP-10 protein. Therefore, MMP-10 may also not be involved in the inhibitory effect of APOM on the proliferation and migration of LC cells.

The transcription of mRNA and the translation of protein are two independent processes which are affected by different factors. Especially in tumour cells, the changing trends of gene mRNA and protein levels are often inconsistent, or even opposite^23^. In vivo, protein is one of the main molecules involved in signal transduction and physiological functions. Therefore, based on the results of this study, we concluded that APOM inhibits the proliferation and migration of LC cells, but may not be related to VDR, NFE2L3 and MMP-10. More research is needed to explore and clarify the real mechanism.

## Supplementary information

Supplementary information.

## Data Availability

All data in this study are presented in this article and its Supplementary Information File.
