# Supplementary material for: Apolipoprotein M inhibits proliferation and migration of larynx carcinoma cells
Source: Sci Rep. 2020 Nov 10;10:19424. doi: 10.1038/s41598-020-76480-w (PMC7655836; doi:10.1038/s41598-020-76480-w)

**Apolipoprotein M inhibits proliferation and migration of larynx carcinoma cells**

Haixiang Xue ^a,1^, Miaomei Yu^b,1^, Ying Zhou^b,1^, Jun Zhang^b^, Qinfeng Mu^b^, Tongbing Chen^c^, Guanghua Luo^b,^**, and Jisheng Liu^d,^*

^a^ Department of Otorhinolaryngology, The Third Affiliated Hospital of Soochow University, Changzhou, Jiangsu Province, China

^b^ Comprehensive Laboratory, The Third Affiliated Hospital of Soochow University, Changzhou, Jiangsu Province, China

^c^ Department of Pathology, The Third Affiliated Hospital of Soochow University, Changzhou, Jiangsu Province, China

^d^ Department of Otorhinolaryngology, The First Hospital Affiliated to Soochow University, Suzhou, Jiangsu Province, China

*****Corresponding author at: 188 Shizi St, Suzhou, Jiangsu Province, China.

E-mail: [ljswwq@sina.com](mailto:ljswwq@sina.com) (J. Liu)

**And**

******Corresponding author at: 185 Juqiang St, Changzhou, Jiangsu Province, China.

E-mail: shineroar@163.com (G. Luo)

**Supplemental Figure S1.** APOM protein levels in vocal cord polyps, LC tissues and matched paracarcinomatous tissues detected by western blot based on capillary electrophoresis technology. A pack with 25 capillary cartridges was used in this study. APOM and β-actin protein levels were detected by 2-40 kDa and 12-230 kDa pre-filled plates, respectively. The blots of patients A and B in Figure 1c in main text are from blots of patients 6 **(b and f.** Indicated by red boxes**)** and 10 **(c and g.** Indicated by red boxes**)** with larynx cancer, respectively. The blots of patients C and D in Figure 1c are from blots of patients 4 and 5 **(a and e.** Indicated by red boxes**)** with vocal cord polyps, respectively. The information from “Other samples” is not relevant to this study.

**Abbreviation** C: Cancer tissue; Pa: Paracarcinomatous tissue; Po: Polyp tissue

**Supplemental Figure S2.** APOM protein levels in TU686 cells detected by Western blot based on capillary electrophoresis technology. A pack with 25 capillary cartridges (12-230 kDa pre-filled plates) was used in this study to detect APOM, β-actin and Lamin B1 protein levels. Panels **a**, **b**, and **c** are the results for electrophoresis of total, cytoplasmic, and nuclear proteins, respectively. For APOM, the loading concentrations of total protein, cytoplasmic protein and nuclear protein were 1.5, 1.5 and 0.65µg/µL, respectively. For β-actin, the loading concentrations of total protein and cytoplasmic protein were 0.1 and 0.2µg/µL, respectively. For Lamin B1, the loading concentration of nuclear protein was 0.2µg/µL.

**Supplemental Figure S3.** Protein levels of VDR, NFE2L3 and MMP-10 detected by traditional Western blot. Panels **a** and **b** are the results for VDR and its associated β-actin, respectively (The molecular weight marker was purchased from Solarbio, China). Panels **c** and **d** are the results for NFE2L3 and its associated β-actin, respectively (The molecular weight marker was purchased from Thermo Scientific, USA). Panels **e** and **f** are the results for MMP-10 and its associated β-actin, respectively (The molecular weight marker was purchased from Thermo Scientific, USA). N1 to N6 are samples of NC group and A1 to A6 are samples of APOM-OE group. Panels **b** and **f** show the results of incubating β-actin antibody after stripping the antibody from the membranes in panels **a** and **e**, respectively. Panels **c** and **d** show the results of cleaving the membrane into two parts according to the molecular weight of the target proteins and incubating with NFE2L3 and β-actin antibodies, respectively. The target bands used in Figure 3 of the main text are indicated by red boxes.

**Supplemental Figure S1**


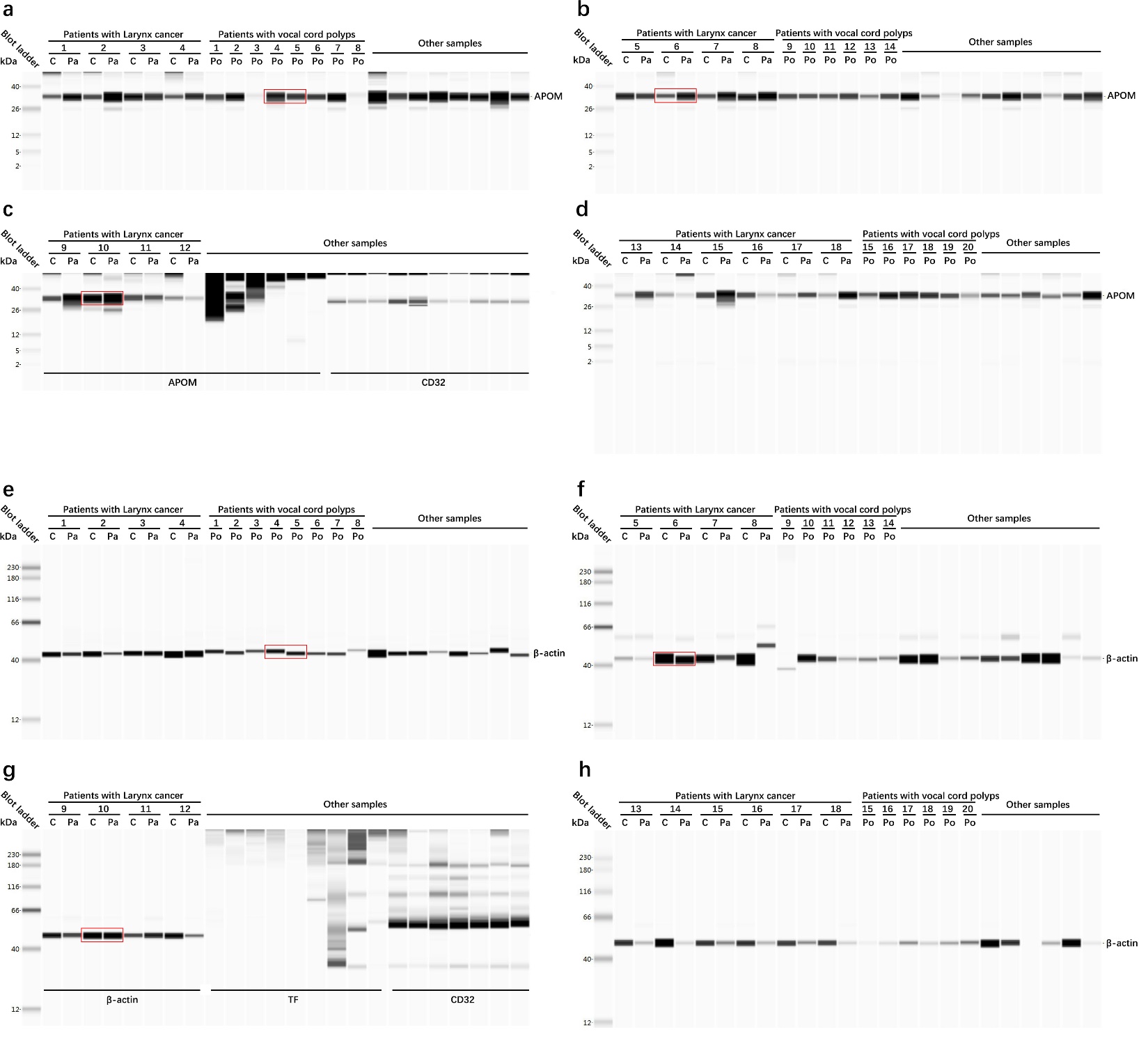


**Supplemental Figure S2**


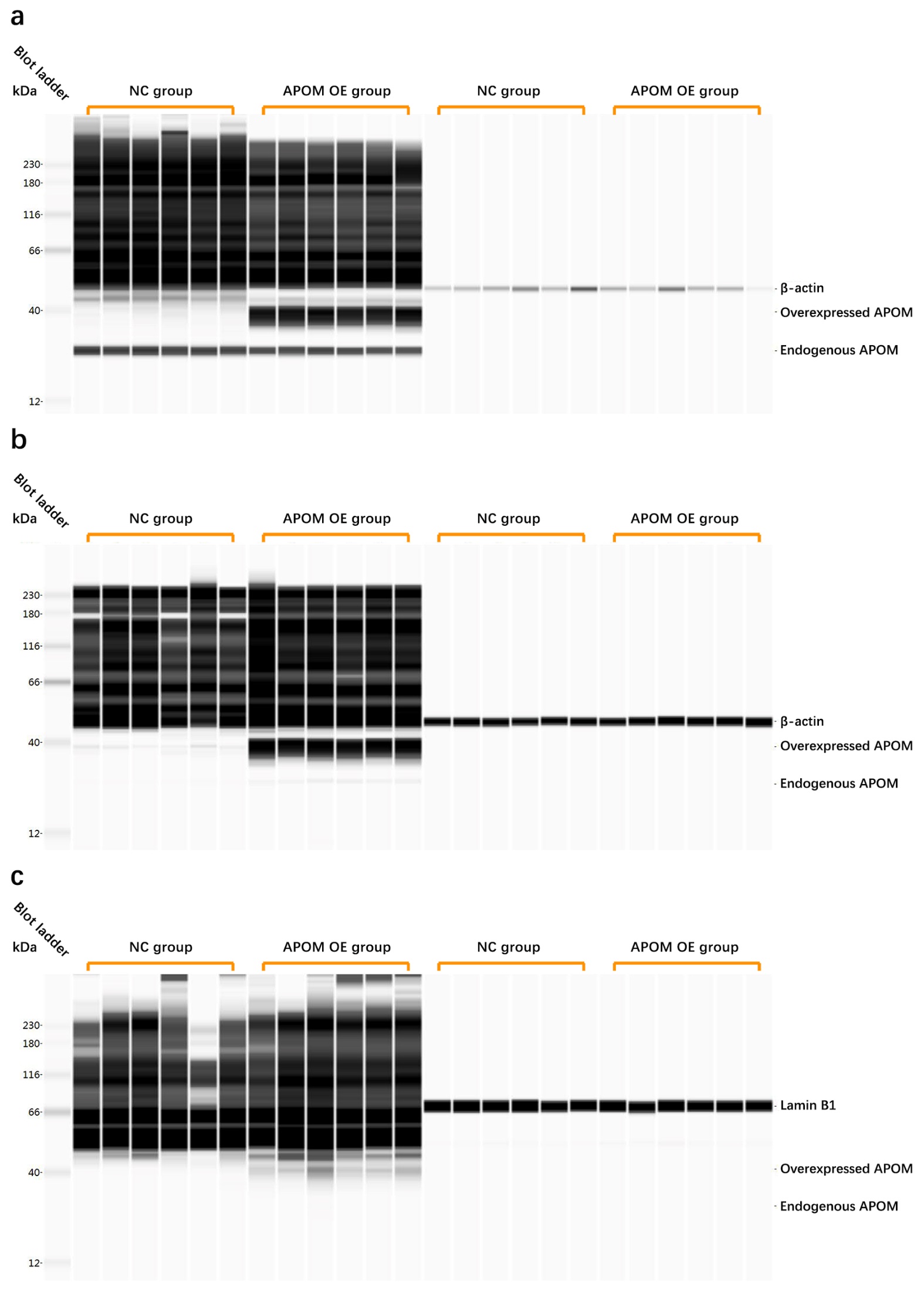


**Supplemental Figure S3**


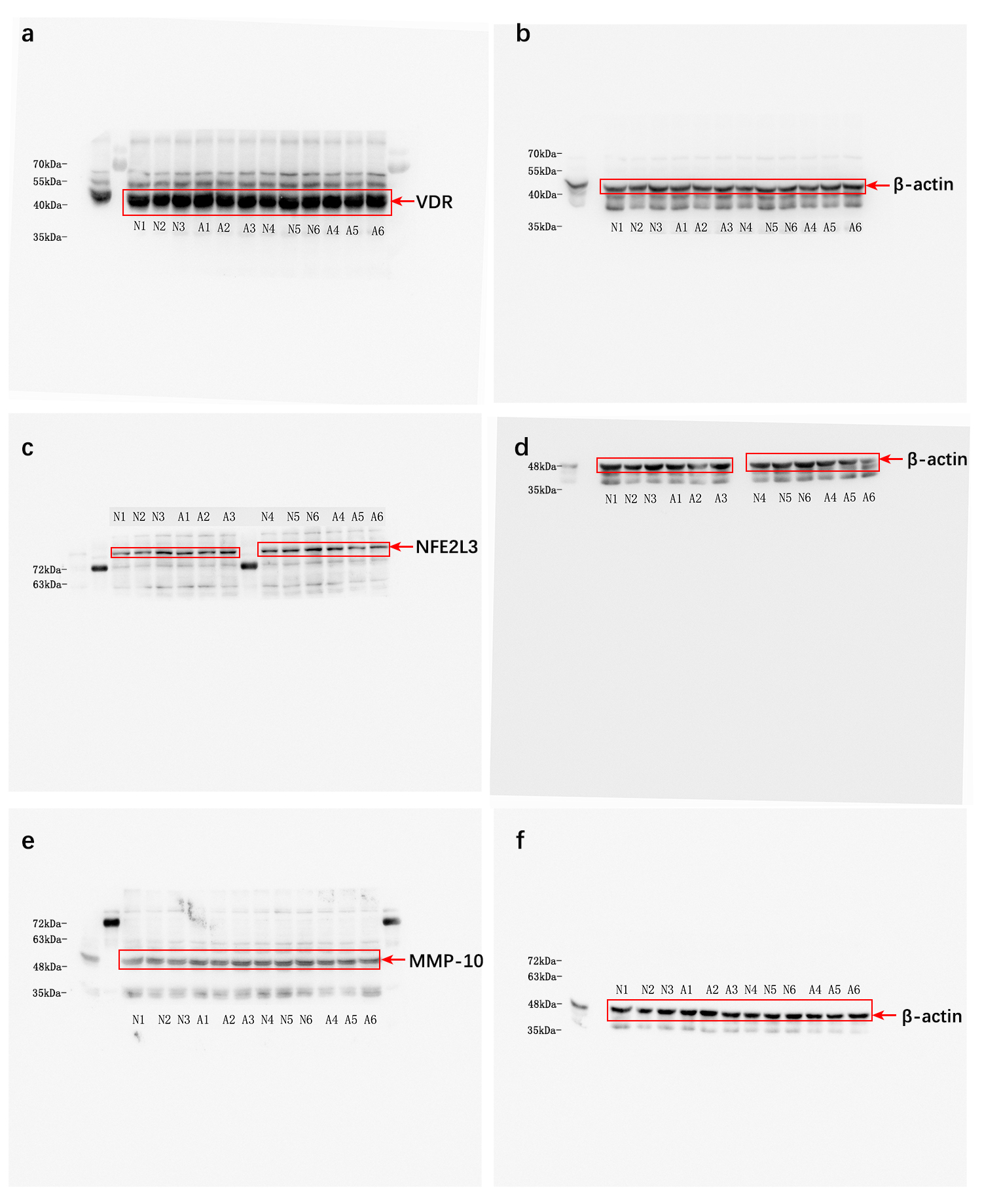

Supplement: Supplementary file 1 — Supplementary information. [file 41598_2020_76480_MOESM1_ESM.docx]
